# 1000-Year Quasi-Periodicity of Weak Monsoon Events in Temperate Northeast Asia since the Mid-Holocene

**DOI:** 10.1038/s41598-017-15566-4

**Published:** 2017-11-09

**Authors:** Kyoung-nam Jo, Sangheon Yi, Jin-Yong Lee, Kyung Sik Woo, Hai Cheng, Lawrence R. Edwards, Sang-Tae Kim

**Affiliations:** 1 Division of Geology and Geophysics, College of Natural Sciences, Kangwon National University, Chuncheon, Korea; 2 Critical zone Frontier Research Laboratory (CFRL), Kangwon National University, Chuncheon, Korea; 3 Korea Institute of Geoscience and Mineral Resources (KIGAM), Daejeon, Korea; 4 Department of Geology and Geophysics, University of Minnesota, Minneapolis, USA; 5 Institute of Global Environmental Change, Xi’an Jiaotong University, Xi’an, China; 6 School of Geography and Earth Sciences, McMaster University, Hamilton, Canada

## Abstract

The Holocene variability in the East Asian summer monsoon (EASM) based on speleothem δ^18^O records has inconsistencies in timing, duration, and expression of millennial-scale events among nearby regions, and even within the same cave. Here, we present another stalagmite δ^18^O record with multi-decadal time resolution from the temperate Korean Peninsula (KP) for the last 5500 years in order to compare with Holocene millennial-scale EASM events from Southeast Asia. Based on our new stalagmite δ^18^O record, millennial-scale events since the mid-Holocene were successfully identified in the KP, representing a noticeable cyclic pattern with a periodicity of around 1000 years. We propose that the Holocene millennial-scale events are common hydroclimatic phenomena at least in the East Asian monsoon system. Meanwhile, the shorter periodicity of millennial-scale events than that of the North Atlantic region is likely to decouple the EASM system from the North Atlantic climate system. This observation suggests that weak EASM and North Atlantic Bond events may have been induced independently by direct solar activity (and then possible feedback) and ocean–ice sheet dynamics, respectively, rather than simple propagation from the North Atlantic to the EASM regions.

## Introduction

To date, changes in the East Asian summer monsoon (EASM) during the Holocene have mainly been reported based on Chinese cave records^1–3^. These contributions have greatly improved our understanding of regional expressions, teleconnections, and possible mechanisms for the long-term monsoonal climate changes in the recent geological past. However, inconsistencies among speleothem δ^18^O-based EASM records from nearby regions^4^ and even within the same cave^5^ have been recognized, particularly in regard to millennial-scale variation. This has hampered the reliability of cave records for high-resolution comparisons^4,6^, and in turn these inconsistencies can be directly connected to the validity of previous paleoclimatic interpretations of millennial-scale EASM variability. Therefore, additional data from other EASM regions are required to establish a hypothesis regarding the phases and timings of Holocene EASM events^5–8^.

Orbital-scale changes in EASM intensity throughout the Holocene show a gradual and consistent decreasing trend^1–8^. This long-term trend in hydroclimatic changes is also a common feature in other monsoonal areas of the Northern Hemisphere (NH), including Indian regions^9,10^ and the Arabian Peninsula^11^. This strongly suggests that regional EASM characteristics are coordinated over the long term^6^, and thus that long-term changes in solar insolation are the main driver of Earth’s monsoon system^1–3^. Meanwhile, millennial-scale weak monsoon events during the Holocene have been reported to have largely different traits^5^. For example, Dark Cave records from southwest China highlight a weak monsoon event at 3.4–3.7 ka^4^, while multiple other studies have indicated that this event is poorly expressed in three stalagmite records from Dongge Cave (DA, D4, and DAS records)^1,2,5^. This weak monsoon event, however, is clearly observed in a record of Heshang Cave (HS-4 record)^3,4^ and possibly Sanbao, and even Qunf caves^11^. As most EASM records are often correlated to North Atlantic ice-rafted debris (IRD) events^2,12^, this weak monsoon event presents a significant challenge to simple teleconnection between high-latitude oceanic and subtropical monsoon regions. Other millennial-scale events (MSEs) recorded in speleothem δ^18^O profiles from Asian monsoon regions have different timings and phases among records^6,7^. Therefore, it remains uncertain whether the speleothem δ^18^O records in EASM regions effectively preserve MSEs during the Holocene. Given that the answer to this question is linked to the validity of Holocene climate variability in global monsoonal systems, this is one of the most necessary topics to understand in Holocene paleoclimatology.

**Figure 1:**
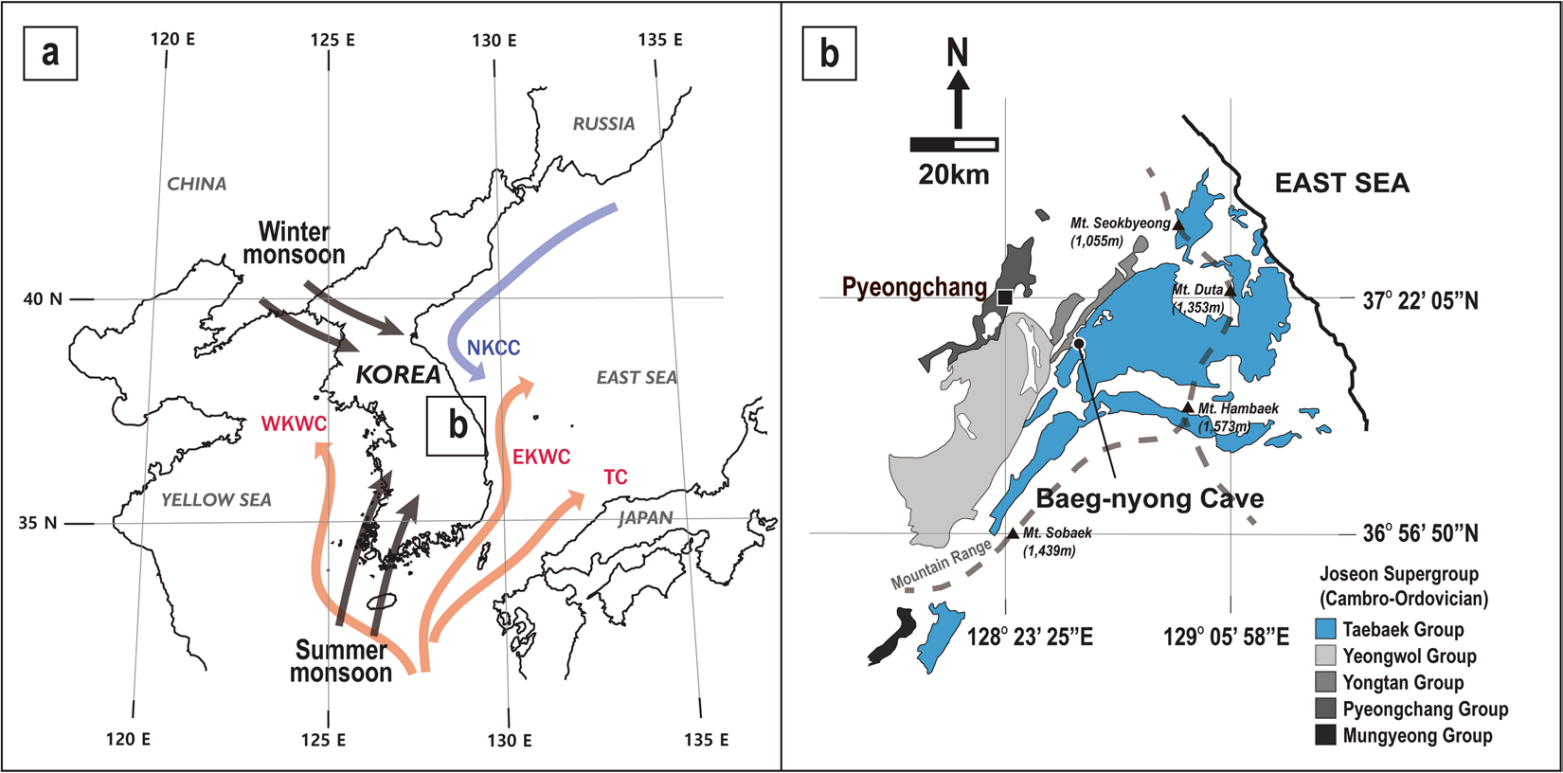
Geographic characteristics of the study area. (**a**) Regional atmospheric and oceanographic settings around the Korean Peninsula, TC = Tsushima current, WKWC = West Korean warm current, EKWC = East Korean warm current, NKCC = North Korean cold current. (**b**) A geological map with the locality of Baeg-nyong Cave and surrounding mountain ranges. These figures were created using the software Adobe illustrator CS6. Base maps for (**a**) and (**b**) are from National Geographic Information Institute (http://www.ngii.go.kr/world/worldmap_en.html). Geological boundaries on (**b**) are based on Korean Institute of Geoscience and Mineral Resources (KIGAM) (https://mgeo.kigam.re.kr/).

To validate previously reported speleothem-based EASM events, the Korean Peninsula (KP) (with an annual temperature of ~12.5 °C and precipitation of ~1250 mm) is an ideal study area because it is located close to subtropical China and corresponds to a typical temperate EASM region, with a different climate regime from those reflected in Chinese records (Fig. 1 and Supplementary Fig. S1)^13,14^. If the MSEs were prominent and general in the East Asian region, the KP likely experienced these hydroclimatic events during the Holocene, although this is not a simple necessary condition. That is, the generality of MSEs could be verified if speleothem records from the KP are closely correlated with previous subtropical EASM records. Baeg-nyong Cave (37° 16′ 19.65″ N/128° 34′ 46.03″ E), from which a speleothem sample was collected for this study, is a typical limestone cave that formed within the Maggol Formation of the Lower Paleozoic Joseon Supergroup located in the central region of the KP (Fig. 1)^14^. Baeg-nyong Cave has been stable environmentally with its depth of over 250 m from the surface and extremely small seasonal variations in the cave interior air temperature and humidity.

Here, we present a new continuous δ^18^O profile with multi-decadal time resolution from a Korean stalagmite sample (BN-1) since the mid-Holocene. We examined the recurrence interval of Holocene EASM events in the Korean peninsula to test its reproducibility from regions outside the Southern China, and performed regional and global comparisons among the paleoclimate records of MSEs to infer both the possible intraregional and interregional linkages during the Holocene.

## Results

**Figure 2:**
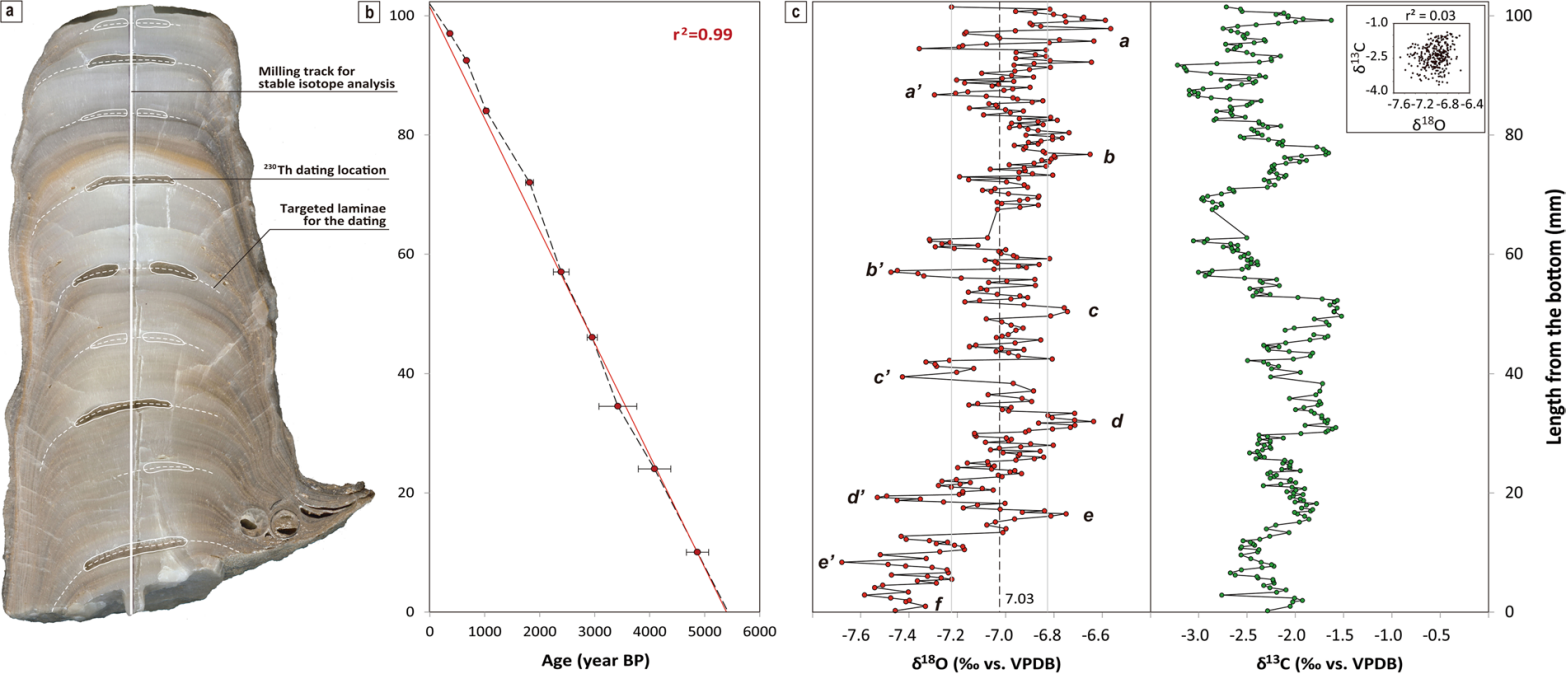
Age dating and stable isotopic results for a stalagmite sample (BN-1). (**a**) A rock slab of BN-1 examined in this study. Also shown are the milling track for stable isotope analysis and ^230^Th dating locations. (**b**) ^230^Th dating results. Dashed lines indicate the age model by simple interpolation between each dating point, while the red line represents a linear age model for the whole section assuming a uniform growth rate. We used the former age model. (**c**) Carbon and oxygen isotopic results. An average value for oxygen isotopes and standard deviation were indicated by a dashed and two gray lines, respectively. The small letters indicate a series of anomalies distinguished by the standard deviation. Also shown in the right panel are carbon isotopic results along with the same y axis to that of an oxygen profile. The insert shows a cross-plot between the oxygen and carbon isotope values.

BN-1 is a gray to white stalagmite that is composed of densely packed calcite crystals without any altered or porous texture, suggesting no post-depositional diagenesis (Fig. 2 and Supplementary Fig. S2). The crystal texture shows fibrous calcites perpendicular to the growth bands. The lower part of BN-1 contains relatively large amounts of detrital sediment including a few silt-sized quartz grains (Supplementary Fig. S3). A large number of clastic sand and silt grains are found lower than 60 mm from the bottom of the sample, with less detrital content above 60 mm depth. There are two visible growth bands with distinct orange and black laminae at around 77.5 and 79.0 mm (Fig. 2) and a black growth lamina at 82.5 mm. Based on a 3-day observation before sampling BN-1, very slow supply rates of cave drip water (<1 drop/h) fed by a soda straw suggest that the stalagmite is not sensitive to short-term extreme weather conditions. Rather, the sample may reflect long-term climatic signals. A year monitoring result in the sampling site shows the temperature range of 12–13 °C and the relative humidity of 99.9% year around (Supplementary Fig. S4), indicating that BN-1 grew under very stable and non-evaporating condition.

Stalagmite BN-1 was dated by ^234^U-^234^U-^230^Th disequilibrium technique, and 5 of total 10 dates have previously been reported^14^ (Supplementary Table S1). The ^238^U concentrations range from 222.2 ± 0.6 to 3242.5 ± 5.7 ppb and the ^230^Th/^232^Th ratios are from 34.9 ± 0.7 to 234.0 ± 5.0 except for an age from the lowermost part which shows a large age error. The ages of 9 subsamples were calculated to be from 4863 ± 201 to 372 ± 32 years ago, respectively, in ascending order from the bottom^14^. The age model for BN-1 was established by interpolations between every ^230^Th dates. Top surface of BN-1 is assumed to the present because it was actively growing at the sampling time. This assumption is reasonable because dating results from the uppermost part between By1–4 and By1–4-U yielded a nearly identical growth rate with that between By1–4-U and top surface which was assumed to be the present (Fig. 2). The bottom age of BN-1 was calculated by extrapolation based on the constant growth rate between the bottommost two dates. The almost completely linear relationship (r^2^ = 0.99) between ^230^Th ages and the stalagmite lengths indicates that our specimen has a very constant growth rate with no significant hiatus.

Subsequently, the stable isotope profile was obtained from 303 subsamples collected at intervals of 0.25–0.75 mm (Fig. 2 and Supplementary Table S2). Based on BN-1 age model, each δ^18^O and δ^13^C value represents the result of isotope fractionation in both the cave interior and outside for 18 years on average. In this study, we primarily focused on the variations in δ^18^O values. Oxygen isotope compositions ranged from −7.68‰ to −6.57‰, with an average of −7.03‰ and a standard deviation of ±0.20‰. These are similar to the values reported previously for recent and fossil speleothems from the KP^13–15^. In addition, the isotopic data did not show any direct relationships with two distinct visible laminae and the lower part of the BN-1 with high levels of detrital components. The pattern of δ^18^O variation could be divided into three orders of changes (Fig. 2). The long-term (first-order) change in δ^18^O is the overall 0.8‰ increase throughout the entire stalagmite; the most apparent middle-term (second-order) variation is characterized by a quasi-cyclic variation on a scale of about 2 cm with δ^18^O changes of 0.60–1.00‰; and the short-term (third-order) variation includes fluctuations on a scale of a few millimeters with δ^18^O changes of about 0.20‰. The second-order excursions have been separated based on the standard deviation and are shown in Fig. 2. A cross plot of δ^18^O versus δ^13^C shows a random distribution with an extremely low correlation coefficient (r^2^ = 0.03), indicating that neither stable isotope was affected by a single controlling factor^16^.

## Interpretation

Previous studies on the temperate KP have not been able to use speleothem δ^18^O records as paleoclimatic evidence mainly because the speleothem samples have shown relatively small δ^18^O changes over time, and the records have been discontinuous and of low resolution^15,17^. However, because the BN-1 δ^18^O record used in this study is continuous for the last 5500 years and has a multi-decadal time resolution (Fig. 2), it provides an opportunity to interpret speleothem δ^18^O records for the KP. Examination of the textural and stable isotope data combined with ^230^Th ages suggested that stalagmite BN-1 was precipitated under a near-isotopic equilibrium state, and that the oxygen isotope signals represent centennial- to millennial-scale paleoclimatic changes. This is because of the calcite crystal morphology showing a typical fibrous texture^18^, an extremely low correlation coefficient between oxygen and carbon isotope values^16^ (Fig. 2), and a high degree of overall similarity with the δ^18^O records of several Chinese stalagmites^19^ (as discussed below). These three lines of evidence indicate a constant and continuous water supply throughout speleothem growth, slow CO_2_ degassing from the water film without strong evaporation, and no strong kinetic isotope effects nor individual geological conditions (i.e., flow path & lithology, etc.), respectively. These interpretations are also consistent with the relatively slow and constant growth rate of BN-1 (Fig. 2).

**Figure 3:**
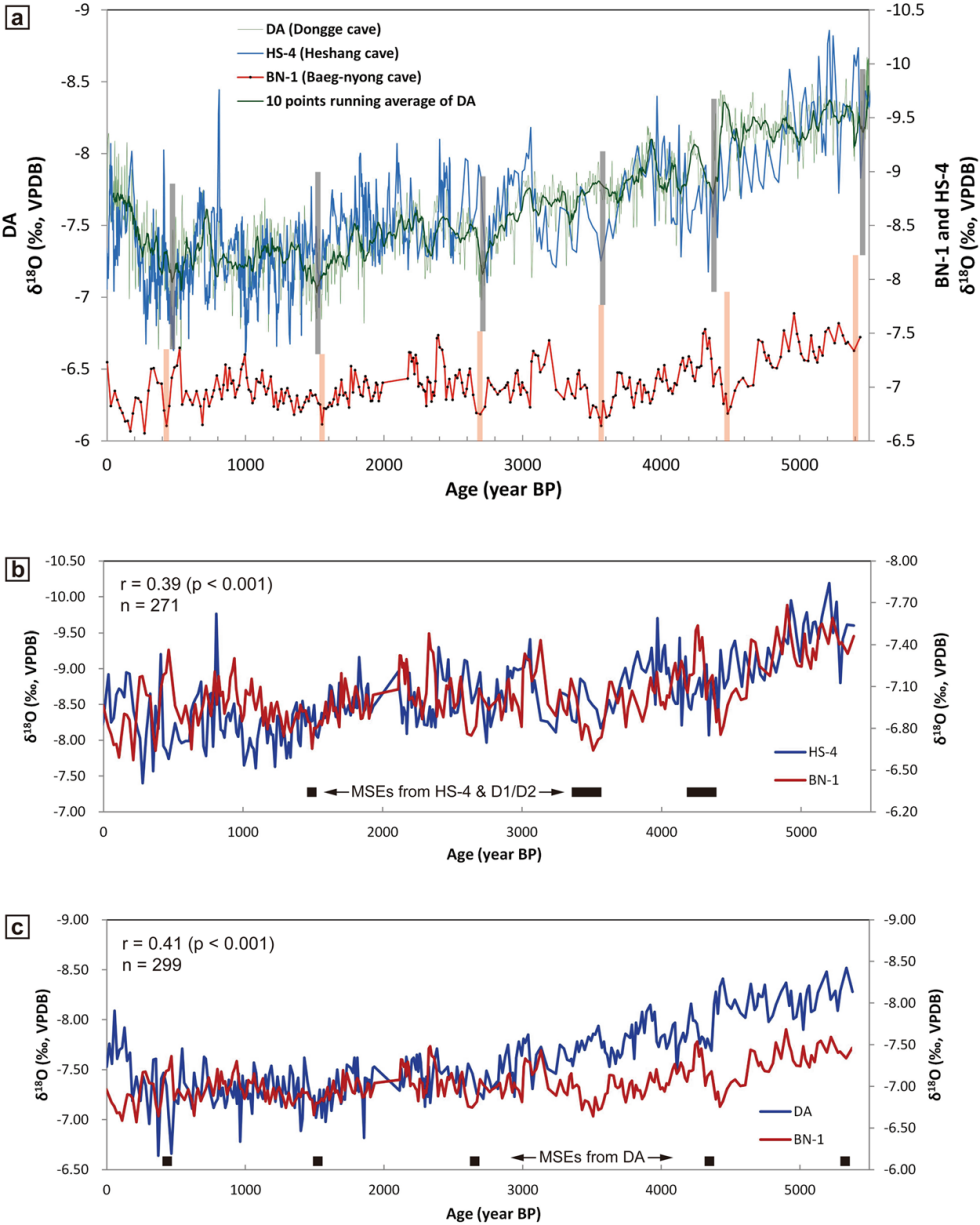
Correlations among Baeg-nyong (BN-1), Dongge (DA) and Heshang (HS-4) records. (**a**) Visible comparison among millennial-scale events (MSEs) represented by BN-1, DA and HS-4 records. Vertical grey bars describe timings of MSEs from plural Chinese records^2–4^. Light orange vertical bars indicate timings of MSEs from BN-1 record. (**b**) Correlation between BN-1 and HS-4 records. Some data subtracted from original datasets to produce similar time-resolution records within 5 years of the maximum age difference between both records. Note that 0.39 of Pearson correlation coefficient was produced between two records. This value was increased when both records are averaged by 3-points running means (Supplementary Fig. S5). Small black rectangles indicate MSEs reported from both of HS-4^3^ and D1/D2 (Dark Cave)^4^ records. (**c**) Correlation between BN-1 and DA records. All processes are same to the case of (**b**). Small black rectangles indicate MSEs reported from DA record^2^.

Although BN-1 record is a single dataset due to the cave conservation program of the nation, comparisons with two major cave δ^18^O records (DA and HS-4 record) from the eastern China show common δ^18^O excursions in the nearly same timings from three records (Fig. 3), suggesting that these isotope excursions had been caused by the similar regional paleoclimatic events in East to Northeast Asia. Pearson correlation coefficients between BN-1 and DA records and between BN-1 and HS-4 records reached to the values of 0.45 and 0.52 (p < 0.001 for both cases), respectively (Supplementary Fig. S5). Thus these regional coeval trends may substitute for the replication examination of BN-1 record in the same cave^19^.

The δ^18^O records from speleothems precipitated under stable isotope equilibrium condition mainly reflects temperature during calcite precipitation and the oxygen isotope compositions of cave drip water^16^. In southern and eastern China, these have been primarily interpreted as changes in oxygen isotope compositions of rainwater^1–5^. Effects of rainfall amount and source changes in water vapor are often considered the most plausible controlling factors for speleothem δ^18^O records from tropical to subtropical Asian monsoon areas^1–5^. However, a stalagmite δ^18^O record from Maboroshi Cave in southwestern Japan has been interpreted as reflecting slightly different controlling factors from cave δ^18^O records from Mainland China because of the different moisture sources for the subtropical Chinese sample and unclear seasonal differences in rainfall δ^18^O values^20^. As these two factors may also affect Korean speleothems, it is necessary to reinterpret the main controlling factors for the δ^18^O record from BN-1 on the KP.

Temperature changes during calcite precipitation alone cannot explain most of the δ^18^O variations observed from BN-1 because ~0.8–1.0‰ changes in the first- and second-order variation in the BN-1 record correspond to temperature decreases of ~4–5 °C (~2/3 of the temperature difference in the full glacial-interglacial periods^14,21^) based on the calcite–water equilibrium oxygen isotope fractionation. Moreover, assuming a net speleothem δ^18^O-mean annual temperature (MAT) relationship of ~0.35‰/°C for the modern mid-latitude regions^22^, the BN-1 record indicates ~2–3 °C warmer conditions since the mid-Holocene, which again conflicts with previous studies from the KP^23^. Although the northeastern Asian sector has often been described as having the most complex seasonal variation in precipitation δ^18^O in the world^24,25^, modern observations indicate that the δ^18^O value of precipitation on the KP is affected by the amount of rainfall in summer and the temperature in winter^26^. Meteoric water δ^18^O records for 2 years from the Chuncheon site^27^ located less than 100 km from the Baeg-nyong Cave also indicate bi-seasonal depletion of ^18^O. This corresponds well to earlier reports that, for the modern mid-latitude regions, the δ^18^O value of mean annual precipitation (MAP) in northeast Asia is controlled by both temperature and the amount of precipitation^28,29^. It is likely that heavy rainfall during the summer rainy season (~60% of MAP) is the main source of groundwater in the KP rather than winter snow (~5%)^29,30^. If this is the case, the effect of the amount of rain during the summer is the major factor controlling the δ^18^O value of the recharge water rather than temperature effect during the winter. An additional factor may be the latitude effect^28^. Because the δ^18^O values of the modern MAP in northeast Asia show decreasing trends with increasing latitude^28^, the δ^18^O values of rainwater at any site are likely to depend on the distance from the sources of Western Pacific moisture (sometimes from the surrounding seas, including the Yellow Sea and East Sea) and moisture recycling. Considering all of the possible factors, we attributed the lower δ^18^O values in the BN-1 record to stronger atmospheric convection in summer between the western Pacific Ocean and Asian continent. Our findings are in line with a previous study that reported a modern speleothem in South Korea that indicates an increasing trend of ~0.22‰ during the modern dry episode^13^. Speleothem δ^13^C values in the KP were mainly interpreted as a proxy of terrestrial primary productivity and in turn changes in both of temperature and precipitation. If this previous interpretation can be directly applied to BN-1 δ^13^C data, very similar second-order variations in both δ^18^O and δ^13^C profiles are likely to highlight our interpretation on BN-1 δ^18^O data (Fig. 2).

## Discussion

The BN-1 record includes three orders of changes that can be translated as gradual changes over the last 5000 years (first order) and two cycles with intervals of about 1000 and 200 years (second and third orders, respectively) (Fig. 2). We focused on the two longer orders of changes considering the time resolution of the BN-1 data. The δ^18^O values of the stalagmite are −7.68‰ to −6.57‰ with an average value of −7.03‰, which is at least ~1‰ higher than any stalagmite δ^18^O records from south to southeast China (Figs 2, 3 and Supplementary Fig. S6). If speleothem δ^18^O values on average are directly affected by distance from the source area and degree of moisture recycling^3,8,9^, this observation suggests that Korean speleothem δ^18^O values reflect a closer source area than those from China (Fig. 1 and Supplementary Fig. S1). Otherwise, it can be inferred that Korean speleothem δ^18^O records are limited to Western Pacific sources, while excluding Indian Ocean components^4,7^. The smaller amplitude and a higher range in δ^18^O values of Korean speleothems than those of Chinese speleothems support this interpretation because the moisture sources from the Western Pacific Ocean might induce more stable and higher δ^18^O values than the far west moisture sources from the Indian Ocean.

**Figure 4:**
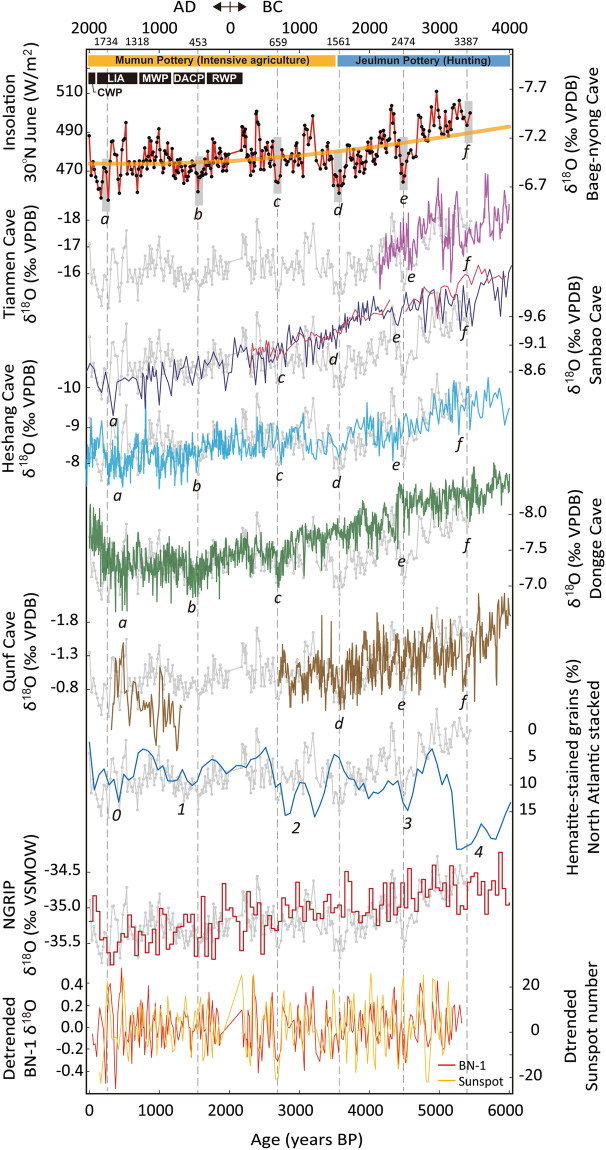
Compilation of speleothem δ^18^O records from Asian monsoon regions, including Tianmen^9^, Sanbao^40^, Heshang^3^, Dongge^2^, and Qunf^11^ caves (30° 55′ N/90° 04′ E, 31° 40′ N/110° 26′ E, 30° 27′ N/110° 25′ E, 17° 10′ N/54° 18′ E, respectively). Also shown are the North Atlantic IRD and NGRIP ice core records. The lowermost graph shows a comparison between the detrended BN-1 record and changes in radiocarbon-based sunspot number. The small letters are the same with those of Fig. 2. Dashed vertical grey lines denote the timings of MSEs represented by BN-1 record. Korean archaeological pottery ages were indicated in top of this figure. CWP = Current Warm Period, LIA = Little Ice Age, MWP = Medieval Warm Period, DACP = Dark Age Cold Period, RWP = Roman Warm Period. The English in this document has been checked by at least two professional editors, both native speakers of English. For a certificate, please see: http://www.textcheck.com/certificate/gBnsul.

The first-order trend in the BN-1 record is comparable to the regional insolation curve (Fig. 4). Most speleothem δ^18^O records from subtropical regions in the NH show consistent increasing trends throughout the Holocene (Supplementary Fig. S6) and this change broadly corresponds to NH solar insolation^1–11^. Our results are in line with those of previous studies in that we found an overall increasing trend in the BN-1 δ^18^O record that has been maintained since the mid-Holocene and corresponds to insolation changes. In the KP, the long-term δ^18^O values of summer rainwater were likely to be gradually increased since the mid-Holocene by weaker atmospheric circulation.

Most recently, Cheng *et al*.^31^ reported “the 2 kyr shift,” which occurred 2000 years ago based on the Dongge Cave δ^18^O (D4) record and various other paleoclimatic records. They suggested that the increasing rate of the Atlantic Meridional Overturning Circulation (AMOC) may be a key process involved in the late Holocene paleoclimatic shift. However, the BN-1 record does not clearly show such a shift, and instead appears to show continuous increasing trend in δ^18^O values, implying weaker EASM circulation during the late Holocene (Fig. 4). Although it remains to be confirmed, this observation may reflect the separation of the temperate climate system from the subtropical climate realm as several Chinese stalagmite δ^18^O records located at latitudes higher than ~30°N^3,8^ show results similar to the BN-1 record (Supplementary Fig. S6).

In the second-order trend, the BN-1 record clearly shows quasi-cyclic MSEs with recurrence intervals of about 1000 years (Fig. 4). These episodes are centered at ~5.4 ± 0.20, 4.5 ± 0.25, 3.5 ± 0.32, 2.7 ± 0.12, 1.5 ± 0.06, and 0.4 ± 0.02 ka BP. The recent two weak monsoon events in our record follow the general climatic divisions called the Current Warm Period (CWP), Little Ice Age (LIA), Medieval Warm Period (MWP), Dark Ages Cold Period (DACP), and Roman Warm Period (RWP), which are not only evident from a wide range of European regions but also various Asian territories^2,12,32^. The BN-1 δ^18^O record from the KP during the LIA is characterized by large fluctuations between wet and dry conditions, possibly linked to a historical record on the number of heavy rainfall events (Fig. 4 and Supplementary Fig. S7). However, these characteristics must be confirmed by further evidence to determine whether it is a local or regional climate phenomenon, otherwise it may simply be due to the low resolution of the record and other factors.

The second-order trend in the BN-1 record emphasizes and generalizes previous MSEs based on various Chinese stalagmite δ^18^O records^2–5^ (Fig. 3). Wang *et al*. (2005) stated that weak EASM events represented from DA stalagmite were centered at 0.5, 1.6, 2.7, 4.4 and 5.5 ka^2^. Hu *et al*. (2008) and Jiang *et al*. (2013) presented that MSEs in HS-4 and D1/D2 stalagmites are apparent with high δ^18^O values at around 0.5, 1.0–1.4, 1.5, 2.8, 3.1–3.7 and 4.1–4.8 ka^3,4^. BN-1 record clearly reproduced such weak EASM events at 0.4, 1.5, 2.7, 3.5, 4.5 and 5.5 ka with a regular interval of 1000 years. In our view, the Heshang Cave record very closely matches the BN-1 record in both the timing and phases of the MSEs^3^. This suggests that the EASM region has been affected by uniform paleomonsoonal changes between wet and dry climate conditions over such a timescale, although individual records include some differences in their exact timing and phases. Whereas, three Dongge Cave records show many inconsistencies in MSEs, even within the same cave^5^. A previous study discussed this problem, and concluded that this discrepancy is due to the different age constraints, including different dating uncertainties, used in different studies; different noise levels, such as hydrological processes; and relatively different and low resolution records, particularly in D4^1,5^.

One of the most striking events can be observed at about 3.5 ka BP in the BN-1 record (Figs 3 and 4). This timing is coincided with the archaeological transitional period from Jeulmun to Mumun Pottery period in the central-southern Korea, which is corresponded to changes in the basic subsistence and social behavior to the intensive agriculture^33^, while the connection between climatic events and cultural impacts needs to be verified based on further evidences. The Dongge records do not show such an event, while it is very obvious in records from other Chinese caves, such as Heshang Cave and Dark Cave^3,4^ (Fig. 4). Because the 3.5 ka event from some Chinese records (HS-4 and D1/D2 records) can be confirmed based on BN-1 record from the KP, it can be concluded that mid to late Holocene cyclicities in EASM were quite regular with the recurrence interval of about 1,000–1,100 years. We further suggest that the early Holocene climate cycle of ~1000 years, such as the 8.2, 9.3, and 10.3 ka events^34–36^, has been sustained in EASM events during the mid to late Holocene. The supplementary event at 3.5 ka in the BN-1 record rearranges the periodicities of Holocene EASM variability. That is, although the MSEs in Holocene EASM changes have been compared to the cycle of about 1500 years revealed by North Atlantic IRD events^2,12^, our data regarding the 3.5 ka event reorganize the previous periodicity of Holocene EASM changes into a quasi-cyclic 1000-year periodicity. This periodicity can be extended to Indian monsoon regions because Qunf δ^18^O record also shows visible excursions in δ^18^O values with a regular interval of about 1000 years from 10 to 3 ka^11^ (Fig. 4).

Based on the results of spectral analyses, our record shows periodicities of 48, 63, and 112 years with a confidence level > 95% and 182, 311, and 1089 years with confidence levels > 80–90% (Supplementary Fig. S8 and Table S3). These cyclicities can be closely compared to several solar cycles such as the Suess cycle (or de Vries cycle; ~180- to 230-year intervals^37^) and the Eddy cycle (~1000-year interval), suggested by total solar irradiance (TSI) and sunspot number records^38,39^. Furthermore, there is a remarkable resemblance with a Pearson correlation coefficient of 0.38 between the detrended BN-1 record and both solar proxy records (Fig. 4; Supplementary Fig. S9). Several Chinese cave records indicate periodicities similar to those of BN-1. For example, Dongge records from three stalagmites describe a ~200-year cycle^2,5^, and other cycles of 65, 180, and 300 years have been reported from the Dark, Heshang, and Sanbao caves, respectively^3,4,40^ (Supplementary Table S3). These lines of evidence indicate that mid-latitude EASM climate is also closely related to solar changes.

In contrast, the notable 1000-year periodicity of the MSEs in the EASM regions is likely to be decoupled from the characteristics of the North Atlantic paleoclimatic changes. The patterns and timings of MSEs including 3.5 ka event clearly indicated by the BN-1 and Chinese stalagmite records has not been observed from previous IRD and Greenland ice core records from the North Atlantic regions^12,41^ (Fig. 4). Our result may support previous wavelet analytical data indicating that a millennial-scale periodicity of Holocene climate changes can be subdivided into a solar cycle of 1000 years and an oceanic cycle of 1500 years^42^. In other words, the Atlantic Meridional Overturning Circulation (AMOC) could modulate a solar cycle of 1,000 years by a relatively slow ocean circulation of 1,500–2,000 years in the North Atlantic regions^43^. It may be possible that the Holocene MSEs revealed by BN-1 record were mainly controlled by solar cycles, while Holocene paleoclimatic events in the North Atlantic regions have been modulated by ocean and ice dynamics. Accordingly, we propose that weak EASM events during the Holocene may have been controlled directly by solar activity rather than via propagation from changes in the North Atlantic climate.

## Methods

### Graphic software

We used the licensed Microsoft Excel, Goldensoftware Grapher & Adobe Illustrator for plotting the graphs and retouching (typing letters and inserting some of marks) of all the figures in this study.

### Age Dating

Ten powder subsamples were collected along the growth axis of the stalagmite BN-1 and were analyzed by U-series disequilibrium techniques for the age determination. Five dating results reported in this manuscript are from our previous study^14^. The ^230^Th dating work was performed at the Minnesota Isotope Laboratory, University of Minnesota in USA and the Isotope Laboratory of Xi’an Jiaotong University in China using multi-collector inductively coupled plasma mass spectrometers (MC-ICP-MS) (Thermo-Finnigan Neptune-*plus*). We use standard chemistry procedures to separate uranium and thorium for dating^44^. A triple-spike (^229^Th–^233^U–^236^U) isotope dilution method was employed to correct for instrumental fractionation and determine U/Th isotopic ratios and concentrations. The instrumentation, standardization and half-lives are reported in refs^45,46^. All U/Th isotopes were measured on a MasCom multiplier behind the retarding potential quadrupole in the peak-jumping mode. We followed similar procedures of characterizing the multiplier as described in ref.^45^. Uncertainties in U/Th isotopic data were calculated offline at 2σ level, including corrections for blanks, multiplier dark noise, abundance sensitivity, and contents of the same nuclides in spike solution. Corrected ^230^Th ages assume the initial ^230^Th/^232^Th atomic ratio of 4.4 ± 2.2 × 10^−6^, the values for a material at secular equilibrium with the bulk earth ^232^Th/^238^U value of 3.8. The U decay constants are reported in ref.^46^.

### Stable Isotope Analysis

For carbon and oxygen isotope analyses of the stalagmite sample (BN-1), subsamples of ~100 µg were drilled along the growth axis with an interval of 0.25 to 0.75 mm using a computer-controlled micro-mill system. The isotopic analyses were performed by McMaster Research Group for Stable Isotopologues (MRSI) at McMaster University, Canada. Measured carbon and oxygen isotope compositions are reported using the common delta (δ) notation relative to the Vienna-PDB (the belemnite from the Pee Dee Formation) standard. The long term analytical errors of NBS 19 for δ^13^C and δ^18^O are 0.04‰ and 0.08‰, respectively.

### Statistical Analysis

Spectral analyses were performed using REDFIT 3.5 to extract the periodicities from the unevenly spaced δ^18^O profile of the BN-1 stalagmite. As in ref.^47^, this program can be used to test if peaks in the spectrum of a time series are significant against the red-noise background from a first-order autoregressive process. The program involves the Lomb-Scargle Fourier transform to overcome unequal time intervals. The input parameters used for the analyses were as follows: nsim = 1000, mctest = T, rhopre = −99.0, ofac = 2, n50 = 4, iwin = 1.

### Data Availability

δ^18^O and U-series data of the BN-1 are included in the Supplementary Materials.

## Electronic supplementary material

Supplementary information
