# Supplementary material for: 1000-Year Quasi-Periodicity of Weak Monsoon Events in Temperate Northeast Asia since the Mid-Holocene
Source: Sci Rep. 2017 Nov 9;7:15196. doi: 10.1038/s41598-017-15566-4 (PMC5680219; doi:10.1038/s41598-017-15566-4)
Supplement: Supplementary file 1 — Supplementary information [file 41598_2017_15566_MOESM1_ESM.pdf]

Supplementary Information for

"1000-Year Quasi-Periodicity of Weak Monsoon Events in Temperate Northeast Asia since the Mid-Holocene"

Kyoung-nam Jo[1,2,*], Sangheon Yi[3], Jin-Yong Lee[1,2], Kyung Sik Woo[1], Hai Cheng[4,5], Lawrence R. Edwards[4], Sang-Tae Kim[6]

[1]Division of Geology and Geophysics, College of Natural Sciences, Kangwon National University, Chuncheon, Korea

[2]Critical zone Frontier Research Laboratory (CFRL), Kangwon National University, Chuncheon, Korea

[3]Korea Institute of Geoscience and Mineral Resources (KIGAM), Daejeon, Korea

[4]Department of Geology and Geophysics, University of Minnesota, Minneapolis, USA

[5]Institute of Global Environmental Change, Xi'an Jiaotong University, Xi'an, China

[6]School of Geography and Earth Sciences, McMaster University, Hamilton, Canada

*Correspondence to kjo@kangwon.ac.kr

Supplementary figures

1. **Figure S1.** Locations of cave records from the Korean Peninsula to the Arabian Peninsula considered in this study
2. **Figure S2.** Photomicrographs of BN-1 composed entirely of fibrous calcite crystals with no evidence of post-depositional diagenesis
3. **Figure S3.** X-ray diffraction (XRD) patterns of BN-1
4. **Figure S4.** Cave monitoring results of temperature and relative humidity in the sampling location for one year
5. **Figure S5.** Correlations among the Korean and major Chinese cave records
6. **Figure S6.** A comparison of continuous cave $\delta^{18}O$ records from the East Asian monsoon regions during the Holocene
7. **Figure S7.** A comparison between the BN-1 $\delta^{18}O$ record and the number of heavy rainfall events documented in a Korean historical literature during the LIA
8. **Figure S8.** Spectral analyses of the BN-1, DA and sunspot number records
9. **Figure S9.** Correlations between the BN-1 and the proxy records for the solar activity

Supplementary tables

10. **Table S1.** ^{230}Th dating results of stalagmite BN-1 from the Baeg-nyong Cave
11. **Table S2.** Oxygen isotopic results of BN-1
12. **Table S3.** Middle to late Holocene periodicities detected from the spectral analyses for BN-1 record and some of major Chinese $\delta^{18}O$ and solar activity records

Supplementary reference list

Figure S1. Locations of cave records from the Korean Peninsula to the Arabian Peninsula considered in this study. The horizontal dashed line in the upper panel indicates 30 °N. Violet arrows indicate schematic outlines of the Indian and East Asian monsoon circulations. A green dashed rectangle is corresponded to an area of the lower panel. The lower panel displays a schematic map of the respective pathways of tropical moisture in the northeastern Asia modified from Liu et al. (2015). This figure was created using the software Adobe illustrator CS6. A base map of the upper panel is from National Geographic Information Institute (http://www.ngii.go.kr/world/worldmap_en.html).

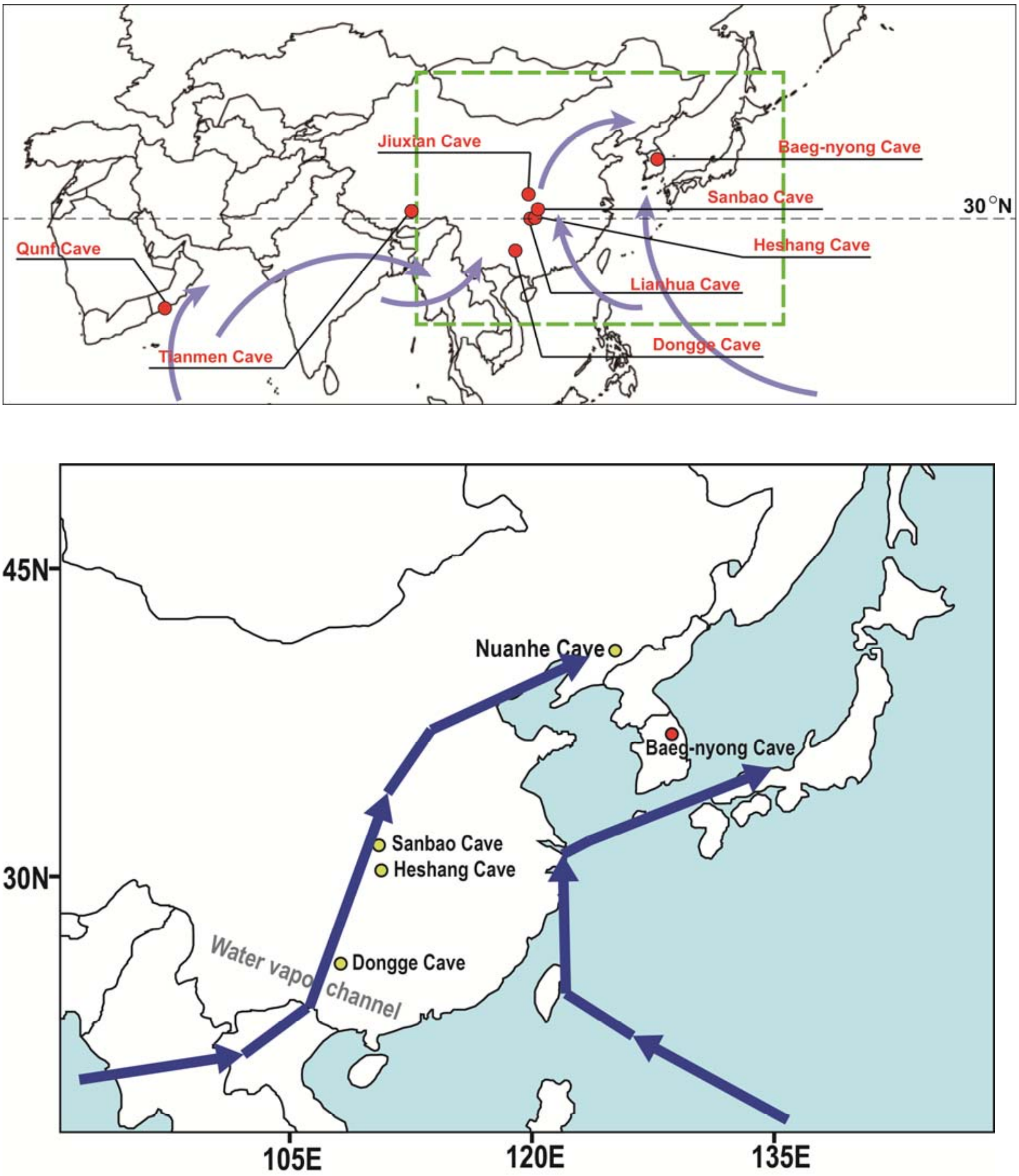

Figure S2. Photomicrographs of BN-1 composed entirely of fibrous calcite crystals with no evidence of post-depositional diagenesis. Large characters D indicate remaining holes after the sub-sampling for U-Th dating. Also shown is the micro-milling track in it. Left: PPL, Right: XPL.

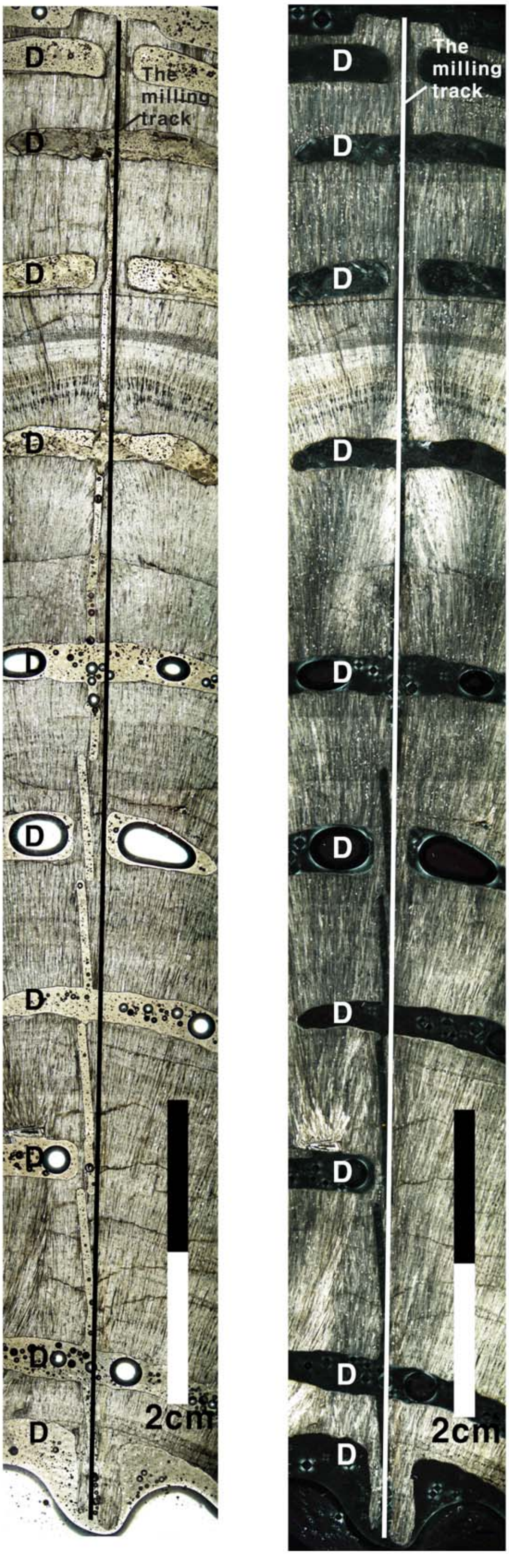

Figure S3. X-ray diffraction (XRD) patterns of BN-1. The pattern from bottom part shows mostly calcite peaks with a minor peak for quartz (a large character Q in the upper panel), while all peaks from middle and upper parts are only for calcite.

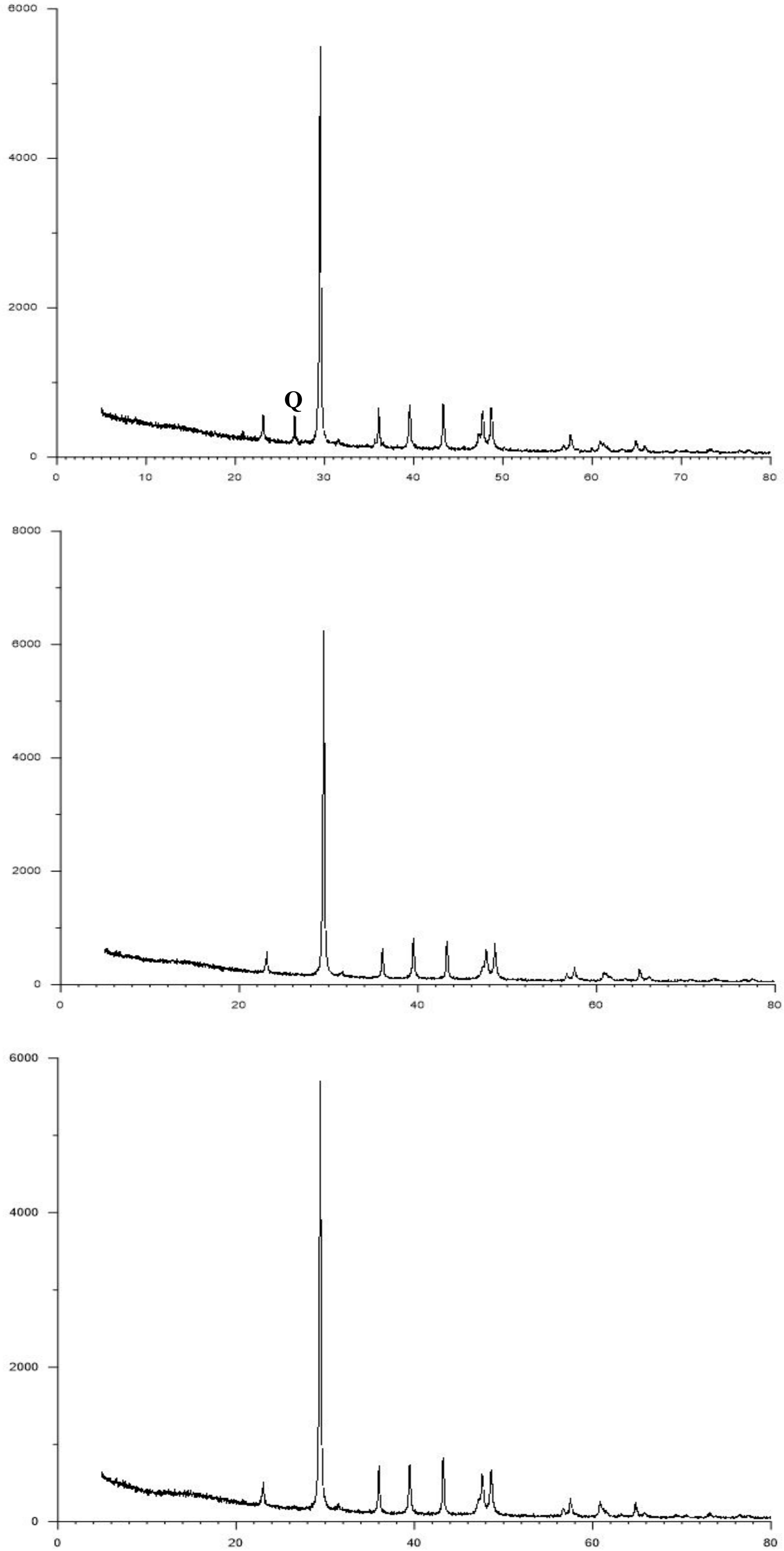

Bottom part

Middle part

Upper part

2θ (degree)

Figure S4. Cave monitoring results of temperature and relative humidity in the sampling location for one year (Woo et al, 2006).

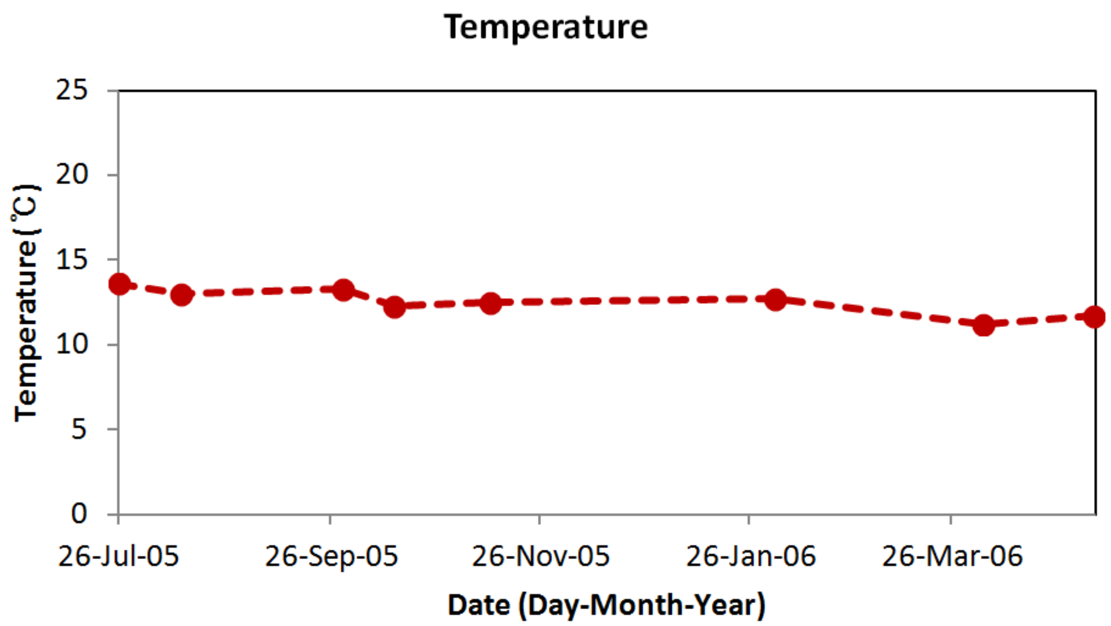

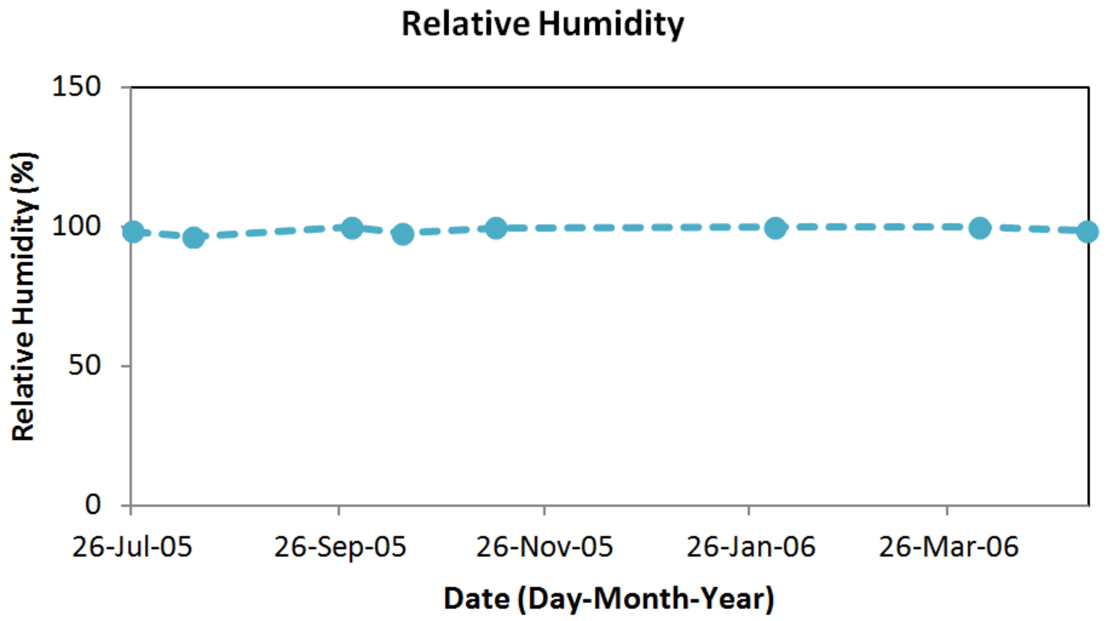

Figure S5. Correlations among the Korean and major Chinese cave records. The upper panel is for a correlation between BN-1 and Heshang (HS-4) $\delta^{18}O$ record, and the lower panel shows a correlation between BN-1 and Dongge (DA) $\delta^{18}O$ records. Simultaneous data from two records were extracted so that both have a same time-resolution of data. The age differences between BN-1, HS-4, and DA records after the extraction are within 5 years. When BN-1 record was correlated with HS-4 record in the upper panel, 3-points running mean data of both records were used and this resulted in a significant value (0.52) of Pearson correlation coefficient. In the lower panel, because the original Dongge cave dataset has a much higher time-resolution than BN-1 record, its 3-points mean data which are corresponded to an almost same time-resolution of BN-1 record were used for this correlation.

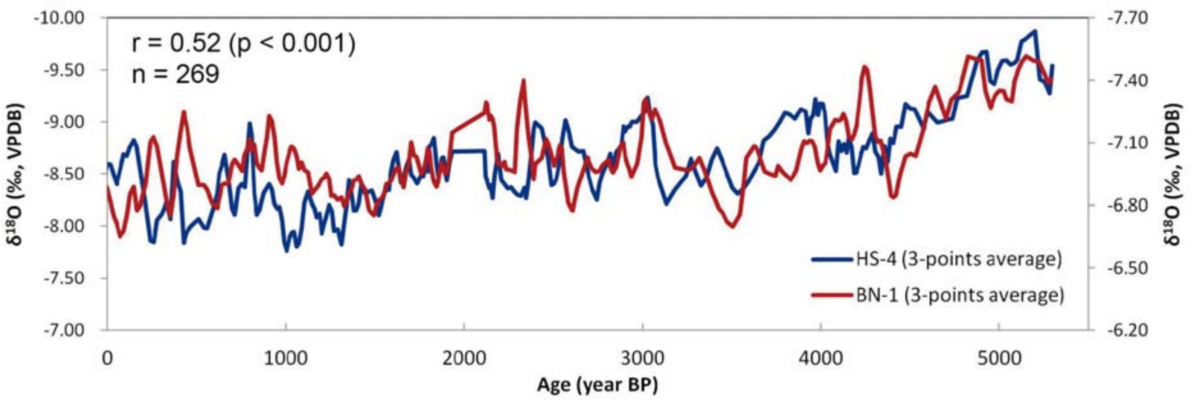

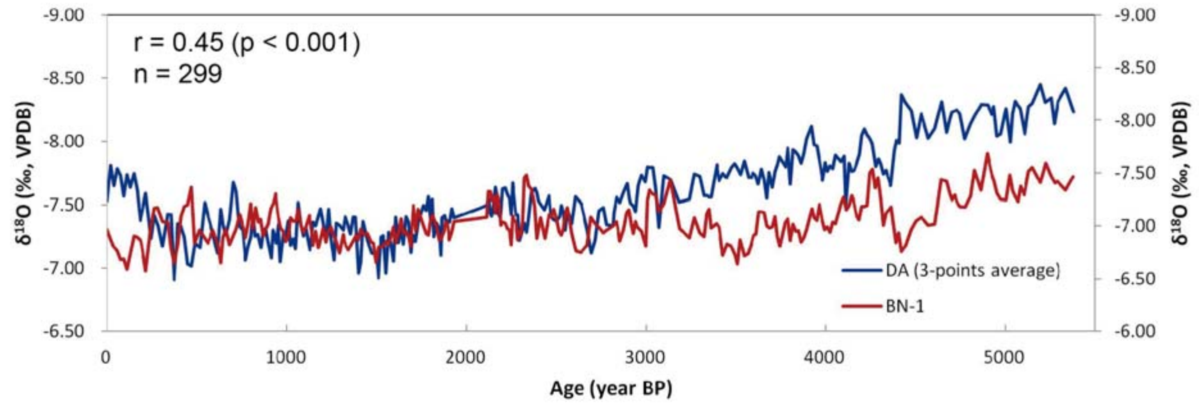

Figure S6. A comparison of continuous cave $\delta^{18}O$ records from the East Asian monsoon regions during the Holocene. Also shown are the millennial-scale EASM events (MSEs; dashed lines marked with a, b, c, d, e, and f) from BN-1 and the correlated points (red arrows) in each record. Please refer to the text and original references for details.

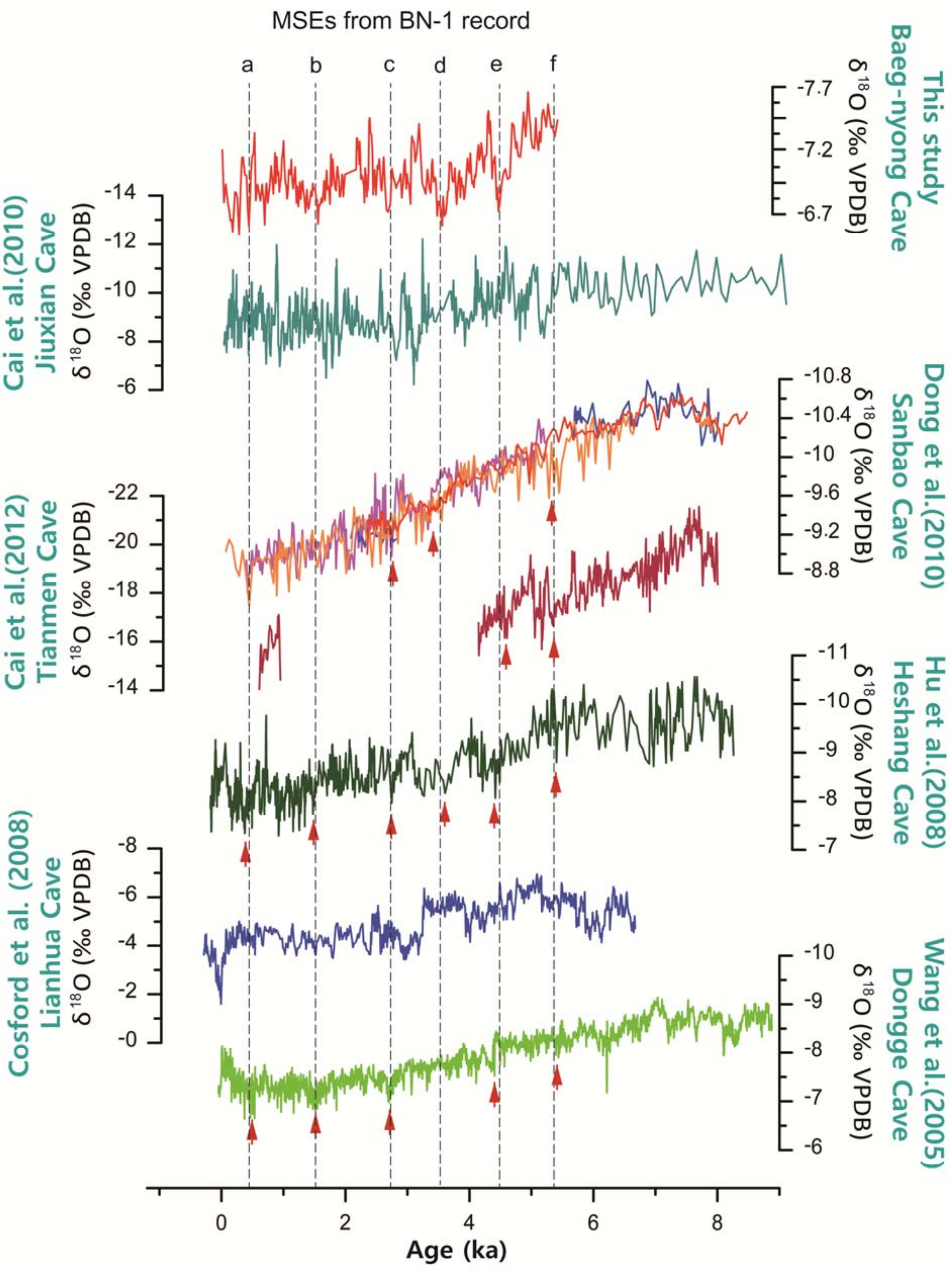

Figure S7. A comparison between the BN-1 $\delta^{18}O$ record and the number of heavy rainfall events during the LIA documented in a Korean historical literature (the Annals of the Joseon Dynasty). For this correlation, BN-1 data were averaged to produce an identical data resolution in both records. A Pearson correlation coefficient reaches a significant value of -0.68 between them.

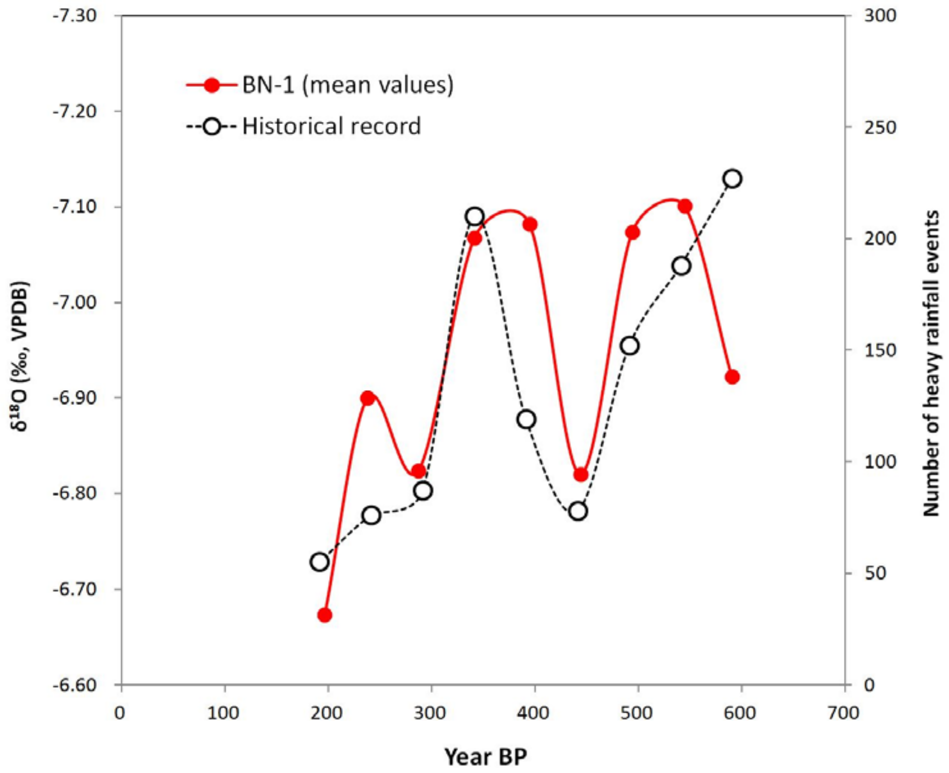

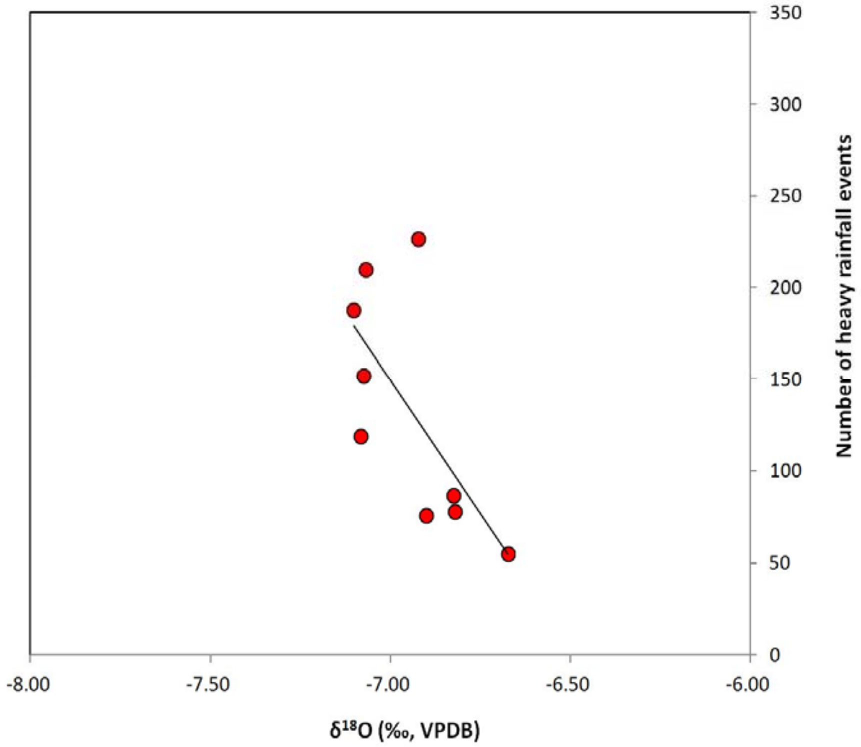

Figure S8. Spectral analyses of the BN-1, DA and sunspot number records. The detected periodicities from these results were presented in Table S3.

TSI

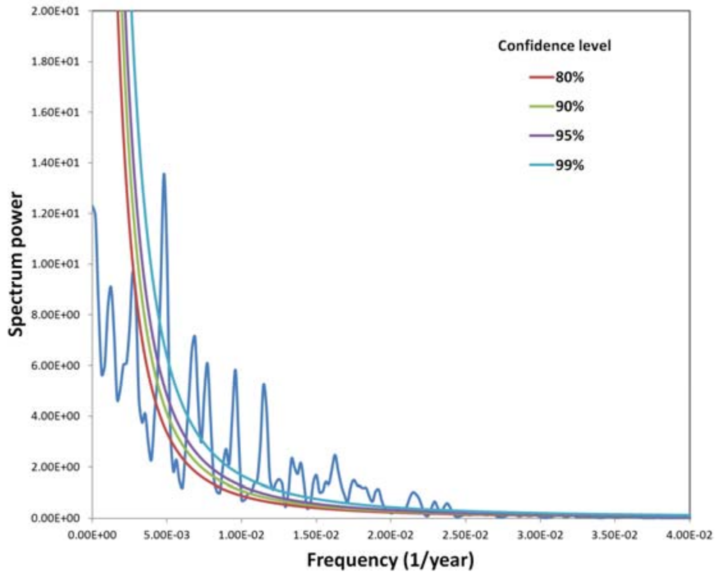

DA

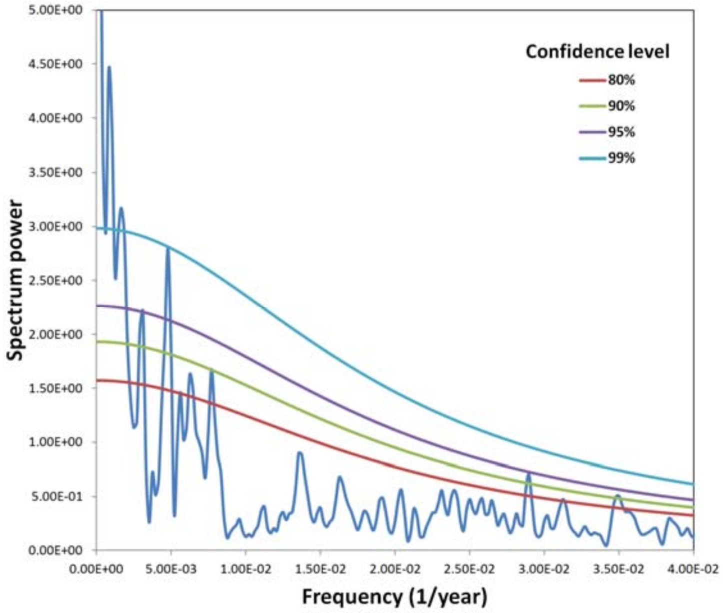

BN-1

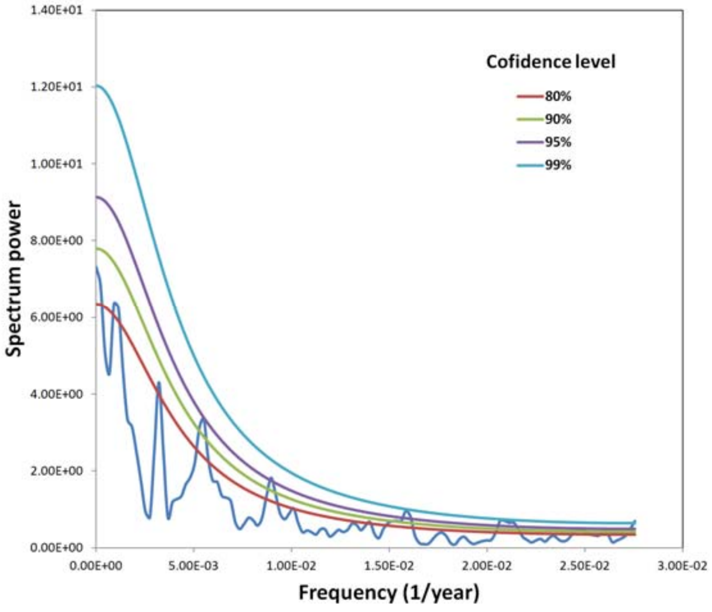

Figure S9. Correlations between the BN-1 and the proxy records for the solar activity. All the data in these correlations were detrended. The results appear the notable similarities between their variabilities. Detrended and smoothed (5-points) datasets of BN-1 and sunspot number records represented a correlation coefficient of 0.38.

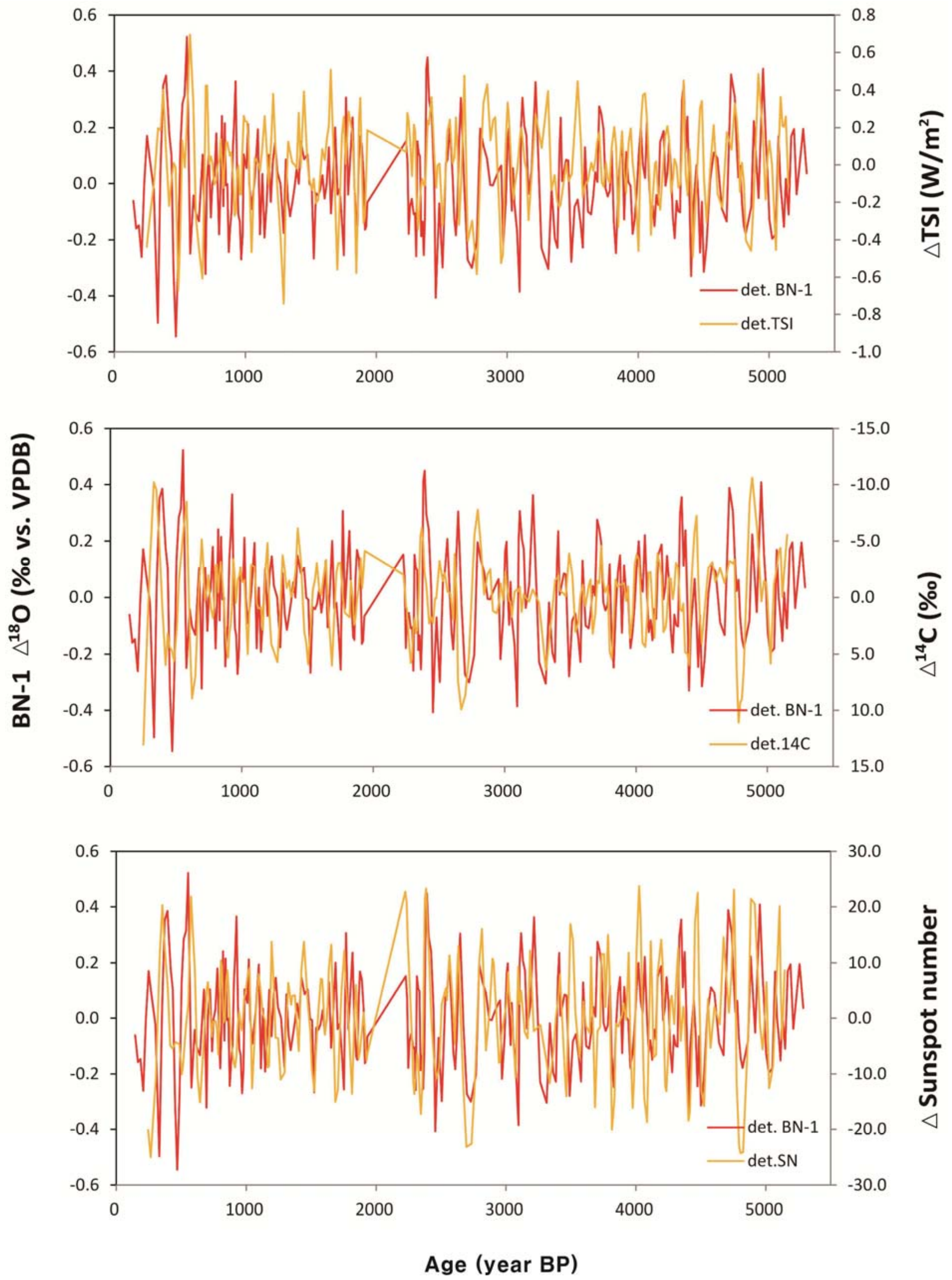

Table S1. ^{230}Th dating results of stalagmite BN-1 from the Baeg-nyong Cave[14].

Sample Number	^{238}U (ppb)		^{232}Th (ppt)		^{230}Th/^{232}Th (atomic ×10^{-6})		δ^{234}U* (measured)		^{230}Th/^{238}U (activity)		^{230}Th Age (yr) (uncorrected)		^{230}Th Age (yr) (corrected)		δ^{234}U$_{Initial}$** (corrected)	
**By1-1-L**	902.2	±10.1	133759	±3058	9	±0	745.5	±10.1	0.0848	±0.0012	5413	±85	**2925**	**±1764**	752	±11
**By1-1**	266.9	± 0.7	7298	± 147	78	± 1.6	1804.1	± 5.7	0.1298	± 0.0005	5144	± 23	**4863**	**± 201**	1829	± 6
**By1-2-L**	3242.5	±5.7	129982	±2610	47	±1	1791.6	±2.7	0.1133	±0.0003	4499	±13	**4084**	**±294**	1812	±3
**By1-2**	222.2	± 0.6	10348	± 209	35	± 0.7	1792.3	± 6.2	0.0985	± 0.0005	3903	± 21	**3419**	**± 343**	1810	± 6
**By-2-L**	303.9	±0.5	3775	±76	104	±2	1796.0	±2.7	0.0781	±0.0002	3082	±9	**2954**	**±91**	1811	±3
**By-2**	296.0	± 0.7	5725	± 115	56	± 1	1811.6	± 3.9	0.0662	± 0.0002	2590	± 10	**2391**	**± 141**	1824	± 4
**By1-3**	308.8	± 1.0	2946	± 60	84	± 2	1800.4	± 7.7	0.0489	± 0.0004	1916	± 16	**1818**	**± 72**	1810	± 8
**By1-4-L**	364.0	±0.8	1966	±40	85	±2	1812.5	±3.4	0.0279	±0.0001	1086	±5	**1031**	**±40**	1818	±3
**By1-4**	319.4	± 0.9	397	± 8	234	± 5	1814.8	± 6.5	0.0177	± 0.0001	687	± 6	**674**	**± 11**	1818	± 7
**By1-4-U**	395.4	±0.7	1698	±34	41	±1	1807.3	±2.7	0.0107	±0.0001	417	±5	**372**	**±32**	1809	±3

The error is 2σ error.
U decay constants: $\lambda_{238} = 1.55125 \times 10^{-10}$ (Jaffey et al., 1971) and $\lambda_{234} = 2.82206 \times 10^{-6}$ (Cheng et al., 2013). Th decay constant: $\lambda_{230} = 9.1705 \times 10^{-6}$ (Cheng et al., 2013).
*$\delta^{234}U = ([^{234}U/^{238}U]_{activity} - 1) \times 1000$. **$\delta^{234}U_{initial}$ was calculated based on ^{230}Th age (T), i.e., $\delta^{234}U_{initial} = \delta^{234}U_{measured} \times e^{\lambda 234 x T}$.
Corrected ^{230}Th ages assume the initial $^{230}Th/^{232}Th$ atomic ratio of $4.4 \pm 2.2 \times 10^{-6}$.
Those are the values for a material at secular equilibrium, with the bulk earth $^{232}Th/^{238}U$ value of 3.8. The errors are arbitrarily assumed to be 50%.

Table S2. Oxygen isotopic results of BN-1.

Age (years ago)	$\delta^{18}O$ (‰, PDB)	Age (years ago)	$\delta^{18}O$ (‰, PDB)	Age (years ago)	$\delta^{18}O$ (‰, PDB)
0	–7.22	842	–7.01	1441	–6.91
28	–6.82	855	–7.20	1455	–6.87
56	–6.96	869	–6.97	1469	–6.96
75	–6.88	883	–7.17	1483	–6.92
94	–6.80	897	–7.03	1504	–6.93
112	–6.76	911	–7.06	1525	–6.85
131	–6.68	925	–6.90	1539	–6.84
150	–6.68	939	–6.97	1553	–6.65
169	–6.59	953	–7.01	1567	–6.80
187	–6.75	967	–7.16	1581	–6.80
206	–6.90	981	–7.21	1594	–6.81
225	–6.89	995	–7.30	1608	–6.85
243	–6.85	1009	–7.08	1622	–6.82
272	–6.57	1023	–6.97	1636	–6.88
300	–6.96	1037	–6.95	1650	–6.98
318	–7.16	1051	–6.85	1664	–6.83
337	–7.17	1065	–6.89	1678	–6.92
365	–7.03	1079	–7.07	1692	–7.06
393	–7.03	1092	–7.04	1706	–6.92
412	–6.78	1106	–7.03	1720	–6.94
431	–6.64	1120	–7.15	1734	–6.89
449	–6.82	1134	–7.00	1748	–6.81
468	–7.08	1148	–6.93	1762	–7.19
487	–7.18	1162	–6.98	1776	–6.95
506	–7.19	1183	–7.09	1790	–7.15
524	–7.36	1204	–6.81	1813	–7.00
543	–6.83	1218	–6.94	1832	–6.92
571	–6.96	1232	–6.79	1846	–6.91
599	–6.88	1246	–6.87	1856	–7.04
618	–6.83	1260	–6.98	1865	–7.10
646	–6.96	1274	–6.84	1875	–7.06
674	–6.82	1288	–6.94	1889	–6.99
688	–6.64	1302	–6.98	1904	–6.86
702	–6.88	1316	–6.91	1913	–6.87
716	–6.97	1330	–6.87	1923	–6.91
737	–6.81	1350	–6.74	1932	–6.94
758	–6.90	1371	–6.92	1942	–7.04
772	–6.96	1385	–6.81	1951	–7.02
793	–7.10	1399	–6.77	1961	–6.86
814	–6.98	1413	–6.81	1975	–6.94
828	–6.88	1427	–6.85	1990	–7.03
2171	–7.07	2751	–7.08	3640	–6.81
2181	–7.32	2774	–7.02	3655	–6.90

2190	–7.31	2796	–6.98	3670	–6.92
2200	–7.23	2819	–6.93	3684	–7.13
2209	–7.26	2836	–6.96	3699	–7.13
2219	–7.11	2871	–6.99	3714	–7.12
2228	–7.29	2882	–7.01	3729	–7.00
2238	–7.21	2894	–7.04	3743	–6.98
2247	–7.00	2911	–6.86	3758	–6.99
2257	–7.03	2934	–6.96	3773	–7.08
2271	–7.02	2951	–7.12	3788	–6.90
2286	–6.97	2962	–7.15	3802	–6.80
2295	–6.95	2974	–7.02	3817	–6.94
2305	–6.82	2985	–6.92	3832	–7.03
2314	–7.08	2996	–7.04	3846	–7.06
2324	–7.04	3008	–6.99	3861	–6.86
2333	–7.03	3031	–6.95	3876	–7.01
2343	–6.86	3054	–6.81	3891	–6.94
2353	–6.95	3065	–7.23	3905	–6.95
2362	–6.91	3076	–7.33	3920	–6.84
2372	–7.05	3094	–7.29	3935	–6.88
2381	–7.45	3111	–7.29	3950	–6.96
2391	–7.47	3128	–7.13	3964	–7.08
2402	–7.36	3156	–7.20	3979	–7.16
2419	–7.34	3191	–7.43	3994	–7.07
2436	–7.18	3242	–6.97	4008	–7.05
2448	–6.88	3299	–6.88	4023	–7.20
2459	–7.00	3328	–7.07	4038	–7.06
2471	–7.07	3356	–6.93	4053	–6.96
2494	–6.88	3379	–6.89	4067	–6.98
2516	–7.10	3396	–7.12	4082	–6.93
2528	–7.08	3408	–7.15	4097	–7.03
2545	–7.15	3427	–6.98	4112	–7.01
2562	–7.03	3449	–7.01	4141	–7.20
2574	–6.94	3463	–6.99	4156	–7.26
2585	–6.91	3486	–6.71	4171	–7.15
2596	–6.98	3508	–6.82	4185	–7.19
2608	–7.11	3530	–6.81	4200	–7.28
2619	–7.17	3552	–6.71	4215	–7.22
2642	–6.92	3567	–6.64	4229	–7.10
2665	–6.76	3581	–6.86	4244	–7.05
2694	–6.74	3603	–6.71	4259	–7.18
2728	–6.81	3625	–6.73	4274	–7.18
4288	–7.19	5209	–7.54		
4303	–7.49	5253	–7.40		
4318	–7.53	5283	–7.58		
4333	–7.35	5312	–7.48		
4347	–7.45	5334	–7.40		
4362	–7.26	5349	–7.41		

4377	–7.00	5393	–7.33
4391	–7.12	5437	–7.46
4421	–7.18		
4436	–7.02		
4450	–6.84		
4465	–6.93		
4480	–6.75		
4502	–6.81		
4531	–6.96		
4561	–7.04		
4590	–7.08		
4627	–7.00		
4664	–7.01		
4701	–7.43		
4730	–7.41		
4745	–7.32		
4760	–7.24		
4774	–7.29		
4789	–7.21		
4804	–7.18		
4833	–7.17		
4855	–7.27		
4885	–7.52		
4922	–7.33		
4959	–7.68		
4981	–7.49		
4995	–7.41		
5010	–7.30		
5032	–7.24		
5062	–7.24		
5084	–7.47		
5099	–7.32		
5113	–7.27		
5128	–7.22		
5143	–7.37		
5165	–7.29		
5187	–7.51		

Table S3. Middle to late Holocene periodicities detected from the spectral analyses[47] for BN-1 record and some of major Chinese $\delta^{18}O$ and solar activity records of the last 6000 years. The different colors in each cell indicate the different values of confidence level for each cyclicity.

	TSI	Sunspot	DA	DAS	HS-4	BN-1
Periodicity	(0-6000)	(0-6000)	(0-6000)	(0-4000)	(0-6000)	(0-6000)
1.2ka			1199			1089
0.6ka			600		605	
0.36ka			369	340		335
0.2ka	209	207	209	200		182
0.15ka	150	149	160			
0.13ka	130	136	133		127	
0.1ka	107	108				**112**
0.08ka	87	84		80	82	
0.07ka	76	76				
0.06ka	62	65				**63**
0.05ka	53	52			56	**48**
0.04ka	42	42			43	**41**
0.03ka			35		29	

99%

95%

90%

80%

Supplementary reference list

Cosford, J. *et al*. East Asian monsoon variability since the Mid-Holocene recorded in a high-resolution, absolute-dated aragonite speleothem from eastern China. *Earth Planet. Sci. Lett.* **275**, 296–307 (2008).

Liu, J. *et al.* Holocene East Asian summer monsoon records in northern China and their inconsistency with Chinese stalagmite $\delta^{18}O$ records. *Earth Sci. Rev.* **148**, 194-208 (2015).

Woo, K. S. *et al.* Scientific investigation of the Baenyong Cave, Pyeongchanggun, Gangwondo, Korea (Korean with a English summary). 85-97 (2006).
